# Bioinformatics identification and validation of m6A/m1A/m5C/m7G/ac4 C-modified genes in oral squamous cell carcinoma

**DOI:** 10.1186/s12885-025-14216-7

**Published:** 2025-07-01

**Authors:** Cheng-Hui Lu, Xue-Lai Yin, Zhuo-Deng Huang, Si-Ang Lv, Jun Wu, Jie Wei

**Affiliations:** 1 Department of Stomatology, Xinhua Hospital, Shanghai Jiao Tong University School of Medicine, Core Unit of National Clinical Research Center for Oral Diseases, Shanghai, 200092 China; 2 Department of Oral Medicine, Shanghai Ninth People’s Hospital, Shanghai Jiao Tong University School of Medicine, College of Stomatology, Shanghai Jiao Tong University, National Center for Stomatology, National Clinical Research Center for Oral Diseases, Shanghai Key Laboratory of Stomatology, Shanghai Research Institute of Stomatology, Shanghai, 200011 China; 3 Department of Oral and Maxillofacial-Head and Neck Oncology, Shanghai Ninth People’s Hospital, Shanghai Jiao Tong University School of Medicine, College of Stomatology, Shanghai Jiao Tong University, National Center for Stomatology, National Clinical Research Center for Oral Diseases, Shanghai Key Laboratory of Stomatology, Shanghai Research Institute of Stomatology, Shanghai Center of Head and Neck Oncology Clinical and Translational Science, Shanghai, 200011 China; 4 Department of Laboratory Medicine, Jiading Branch of Shanghai General Hospital, Shanghai Jiao Tong University School of Medicine, Shanghai, 201803 China; 5 Department of Pathology, The Affiliated Hospital of Youjiang Medical University for Nationalities, Key Laboratory of Molecular Pathology in Tumors of Guangxi Higher Education Institutions, Baise, 533000 China

**Keywords:** Oral squamous cell carcinoma, Bioinformatics analysis, Biomarkers, Prognostic signature, RNA modification

## Abstract

**Background:**

RNA modifications, including m6A, m1A, m5C, m7G, and ac4C, may play a role in the occurrence and development of cancer, such as proliferation. However, the effects of RNA modification-related genes (RRGs) in the development of oral squamous cell carcinoma (OSCC) have not been fully elucidated. The present study aimed to evaluate the effects and mechanisms of RRGs on OSCC development progression.

**Methods:**

RNA-seq transcriptome data, along with clinical and prognostic information, were extracted for 328 patients with OSCC from the TCGA database. A total of 49 RRGs were analyzed for differential expression. We then performed Lasso analysis, as well as univariate and multivariate Cox regression analyses, followed by Kaplan-Meier survival analysis to identify relevant prognostic genes and establish a risk-prognosis model. Patients were categorized into high-risk and low-risk groups, and gene set enrichment analysis (GSEA) was conducted to analyze differences in gene signatures between these two groups, using data from the Kyoto Encyclopedia of Genes and Genomes (KEGG) and Gene Ontology (GO) databases. RT-PCR was employed to validate the expression levels of differentially expressed genes in OSCC samples. The four most significantly differentially expressed genes were selected for further functional analysis, and small interfering RNA (siRNA) vectors targeting these genes were transfected into OSCC CAL27 cells. The Cell Counting Kit-8 (CCK-8) assay was used to evaluate cell proliferation. Additionally, a subcutaneous CAL27 xenograft model transfected with short hairpin RNA (shRNA), combined with Ki-67 immunohistochemical (IHC) staining and TUNEL assay, was used to investigate their underlying molecular mechanisms in vivo.

**Results:**

Among the 49 RRGs, four genes (IGF2BP2, HNRNPC, NAT10, and TRMT61B) were found to be associated with the development of OSCC. Based on various methodological validations, a risk score model was constructed using these four genes. The high-risk and low-risk groups of OSCC patients exhibited significantly different survival outcomes and clinicopathological characteristics. Patients in the low-risk group had longer overall survival (OS) and lower mortality rates compared to those in the high-risk group. The nomogram and decision curve analysis (DCA) demonstrated that our risk model accurately and reliably predicted the impact of risk factors on OS at 1-, 3-, and 5-year. Additionally, risk scores correlated with the infiltration of several immune cells, particularly CD8^+^ T cells and B cells, which showed significant negative correlations. Furthermore, the results of the CCK-8 assay indicated that inhibition of NAT10 and IGF2BP2 expression using siRNA inhibited the proliferation of OSCC cell lines in vitro. Meanwhile, inhibition of NAT10 and IGF2BP2 expression using shRNA influenced proliferation of tumorigenicity in vivo.

**Conclusion:**

In this study, we established a risk model and nomogram based on four RRGs, which can be used for risk stratification and predicting survival outcomes in patients with OSCC. This provides a reliable reference for individualized therapy in OSCC patients.

**Supplementary Information:**

The online version contains supplementary material available at 10.1186/s12885-025-14216-7.

## Introduction

Globally, oral squamous cell carcinoma (OSCC) is the most prevalent form of oral cancer, accounting for over 90% of all oral malignancies and ranking among the top 10 cancers [1]. Due to its ability to metastasize and disrupt the functions of the upper gastrointestinal and respiratory systems, OSCC is associated with poor survival outcomes and high rates of deformity [2]. Early detection of OSCC is challenging, as the disease often does not cause discomfort or noticeable symptoms in its early stages. Consequently, most patients are diagnosed during routine oral examinations or only after the disease has progressed to advanced stages [3]. Although advances in surgery, radiation, and integrated multidisciplinary therapy have improved the prognosis for OSCC patients, their 5-year survival rates remain low [4]. While the 5-year survival rate for early-stage OSCC is approximately 80%, it drops to only 20% for advanced OSCC [5]. Clinically, OSCC poses significant medical and social challenges in many countries and represents a considerable health burden for individuals.

The primary method used in clinical practice to assess the prognosis of OSCC is TNM staging. However, additional molecular diagnostics should be considered for early detection. Understanding the molecular mechanisms that drive OSCC progression can aid in developing effective treatment options. Furthermore, assessing the pathological development and identifying effective early prognostic biomarkers for OSCC will enhance predictions of overall survival (OS) outcomes and guide clinical practice. RNA modifications, particularly methylation and acetylation, are important processes in epigenetic regulation and show promise as biomarkers. These modifications are involved in various biological and pathological processes and play critical roles in tumor development, proliferation, invasion, and metastasis [6–10]. More than 70 types of RNA methylation modifications have been identified, with N6-methyladenosine (m6A) [11–14], 5-methylcytosine (m5C) [15], N1-methyladenosine (m1A) [16], and 7-methylguanosine (m7G) [17] being the most significant [18, 19]. The only acetylation modification demonstrated to regulate mRNA stability, processing, and translation is N4-acetylcytidine (ac4C), a highly conserved RNA modification whose dysregulation may be linked to the growth of various tumors [10].

The significance of risk models based on m6A/m5C/m7G and m6A/m1A/m5C/m7G-related genes in predicting molecular subtypes and prognosis has been shown for pancreatic cancer (PC) [20] and colon cancer (CC) [21], respectively. Recent studies have constructed risk models using m6A/m1A/m5C-related genes to predict overall survival and prognosis in head and neck squamous cell carcinoma (HNSCC) [22] and hepatocellular carcinoma (HC) [23]. Another study included m6A/m1A/m5C/m7G/m6Am/Ψ-related genes in a prognostic model, finding significant associations with immune cell infiltration, gene mutations, and survival outcomes in OSCC patients [24]. However, the roles of the four main RNA methylation modifications (m6A, m1A, m5C, and m7G) and the acetylation modification (ac4C) in predicting overall survival and prognosis for OSCC have not been definitively established.

Continued exploration is essential to uncover RNA modification-related genes (RRGs), particularly those associated with m6A, m1A, m5C, m7G, and ac4C RNA modifications in OSCC development, which remain largely undiscovered. The Cancer Genome Atlas (TCGA) and Gene Expression Omnibus (GEO) databases provide a vast amount of publicly available RNA-seq transcriptome data. In this study, the expression of RRGs in OSCC and their effects on overall survival were investigated. Additionally, an external validation cohort and functional experiment were used to confirm its prognostic value. Our study may enhance our understanding of the mechanisms underlying OSCC progression and provide insights into potential individualized treatments for OSCC.

## Materials and methods

### Collection of TCGA-OSCC cohort data

This study was conducted in accordance with the Declaration of Helsinki (revised in 2013). Human data were sourced from The Cancer Genome Atlas (TCGA) database, which provides publicly accessible, anonymized clinical and genomic datasets. Specifically, raw transcriptome sequencing data and corresponding clinical annotations for oral squamous cell carcinoma (OSCC) patients were retrieved from the TCGA-HNSC project ( https://portal.gdc.cancer.gov/projects/TCGA-HNSC), ensuring compliance with privacy regulations and ethical standards for secondary data analysis. All data were accessed on May 1, 2023. Gene expression profiles were downloaded in the “HTSeq-FPKM” format, ensuring consistent data processing. Only samples originating from the oral cavity (e.g., tongue, floor of mouth, buccal mucosa) and possessing complete survival information were retained for subsequent analyses. Data were filtered using the following exclusion criteria: (1) non-oral squamous cell carcinoma cases (e.g., laryngeal, hypopharyngeal, oropharyngeal, lip, larynx, hypopharynx, or tonsillar sites), (2) missing survival follow-up data, or (3) incomplete/inconsistent TNM staging information. Only OSCC cases originating from oral cavity subsites-including the alveolar ridge, base of tongue, buccal mucosa, floor of mouth, hard palate, and oral tongue-were retained. Ultimately, a total of 328 oral squamous cell carcinoma (OSCC) patients with transcriptome sequencing and survival data were selected for further analysis (Supplementary Table S1). The extracted clinical information included sex, age, TNM classification, and survival outcomes. Among the 328 patients, 155 were under 60 years old, while 173 were over 60. There were 102 female patients and 226 male patients. In terms of tumor staging, 122 patients were classified as T1 or T2, while 196 patients were classified as T3 or T4; the staging for 10 patients was unknown. Additionally, 223 patients were classified as N0, 91 as N1, and 14 as N2 or N3, with the status of other patients being unknown. Finally, there were 309 patients classified as M0, 2 patients as M1, and 17 patients with unknown M stages (Table 1).

**Table 1:** Clinic pathological characteristics of patients with OSCC

Characteristic	No. of cases	%
Age, years (%)		
≤60	155	47.3
>60	173	52.7
Gender (%)		
Female	102	31.1
Male	226	68.9
n	328	
T stage, n (%)		
T1	18	5.5
T2	104	31.7
T3	82	25
T4	114	34.8
Unknown	10	3
N stage, n (%)		
N0	167	50.9
N1	56	17.1
N2	88	26.8
N3	3	0.9
Unknown	14	4.3
M stage, n (%)		
M0	309	94.2
M1	2	0.6
Unknown	17	5.2

In this study, certain clinical variables (e.g., T, N, and M staging) were missing for a subset of patients. Specifically, T staging was unavailable for 10 patients, N staging was unavailable for 14 patients, and M staging was missing for 17 patients (out of 328 total). Before performing the primary analyses, we evaluated the proportion and potential patterns of missingness for these variables. Since the overall rate of missing data was relatively low, and our preliminary assessment suggested data were missing at random (MAR), a complete-case analysis was conducted for multivariate regression whenever feasible. In other words, patients lacking relevant variables were excluded from those specific analyses to minimize imputation bias.

### RNA sequence analysis and RRGs

The R software (R, URL http://www.R- project. org, R version 4.0.5) was used to identify differentially expressed long non-coding RNAs (lncRNAs) between OSCC and normal tissues. A total of 49 RNA modification-related genes (RRGs) related to m6A, m5C, m1A, m7G, and ac4C were extracted from the TCGA databases. This included expression data on writers (METTL3, METTL14, METTL16, WTAP, KIAA1492, RBM15, RBM1, ZC3H13, NOP2, NSUN2, NSUN3, NSUN4, NSUN5, NSUN7, DNMT1, TRDMT1, DNMT3 A, DNMT3B, TRMT6, TRMT61 A, TRMT61B, TRMT10 C, BMT2, RRP8, METTL1, WDR4, WBSCR22, TRMT112, NAT10), readers (YTHDC1, YTHDC2, YTHDF1, YTHDF2, YTHDF3, IGF2BP1, IGF2BP2, IGF2BP3, HNRNPA2B1, HNRNPC, HNRNPG, RBMX, FMR1, LRPPRC, ALYREF), and erasers (FTO, ALKBH5, TET2, YBX1, ALKBH1, ALKBH3) (Table 2). Differential expression analysis was performed using the R program (*p* < 0.05). Heatmaps and boxplots were constructed using the ‘ggplot2’ package in R.

**Table 2:** RRGs of m6 A/m5 C/m1 A/m7G/Ac4 C

RNA modification	Writer	Reader	Eraser
m6A	METTL3, METTL14, METTL16, WTAP, KIAA1492, RBM15, RBM1, ZC3H13	YTHDC1, YTHDC2, YTHDF1, YTHDF2, YTHDF3, IGF2BP1, IGF2BP2, IGF2BP3, HNRNPA2B1, HNRNPC, HNRNPG, RBMX, FMR1, LRPPRC	FTO, ALKBH5
m5C	NOP2, NSUN2, NSUN3, NSUN4, NSUN5, NSUN7, DNMT1, TRDMT1, DNMT3 A, DNMT3B	ALYREF	TET2, YBX1
m1A	TRMT6, TRMT61 A, TRMT61B, TRMT10 C, BMT2, RRP8	YTHDF1, YTHDF2, YTHDF3, YTHDC1	ALKBH1, ALKBH3
m7G	METTL1, WDR4, WBSCR22, TRMT112		
ac4C	NAT10		

### Construction and evaluation of the four-RRGs prediction model

To identify prognostic genes and construct the risk model, we first performed differential expression analysis of the training cohort using the ‘limma’ package (R 4.0.5; limma v3.20.5), defining differentially expressed genes (DEGs) as those with an adjusted *p*-value (FDR) < 0.05 and |*log*_2_-fold change (*log*_2_FC)| > 1.0. Each DEG was then screened via univariate Cox proportional hazards regression to evaluate its correlation with overall survival (OS). Genes with *p* < 0.05 in this analysis were designated as candidate prognostic factors. These candidates were refined using LASSO Cox regression (via ‘glmnet’ package v4.1–6), which applies a penalty to the absolute size of regression coefficients, shrinking some coefficients to zero and selecting the most robust markers. The final four genes (IGF2BP2, HNRNPC, NAT10, TRMT61B) identified by LASSO were used to construct a risk score model. For each patient, the risk score was calculated as: Risk Score = ∑ (β_i_ × Gene_i_), where β_i_ represents the Cox coefficient of the i-th gene, and Gene_i_ is the normalized expression level of that gene. Using the median risk score as cutoff, patients were stratified into high-risk (≥ median, n = 164) and low-risk (< median, n = 164) subgroups within the 328 TCGA OSCC cohort. This stratification enabled further survival analysis and evaluation of clinicopathological correlates. Differentially expressed genes between groups were subjected to Gene Set Enrichment Analysis (GSEA) to identify enriched pathways. Prognostic performance was validated via multivariate Cox regression and receiver operating characteristic (ROC) analysis, with the signature’s predictive power confirmed in an independent cohort using identical methodologies. Statistical significance was defined as *p* ≤ 0.05.

### Cell culture in vitro and siRNA transfection

OSCC cell lines CAL27, WSU-HN30, WSU-HN6, SCC9, and human epidermal keratinocyte cell line HaCaT were obtained from the American Type Culture Collection (ATCC, Virginia, USA). The cells were cultured in RPMI-1640 medium (Gibco, Massachusetts, USA) supplemented with 10% fetal bovine serum (FBS) and 1% penicillin/streptomycin (Gibco, Thermo Scientific, New York, USA) in a 5% CO_2_ atmosphere at 37 °C. Cells were passaged every 2–3 days when they reached 70–80% confluency.

For gene knockdown experiments, we purchased three independent siRNAs per target gene (IGF2BP2, HNRNPC, NAT10, TRMT61B) from Sangon Biotech (Shanghai, China). CAL27 cells were seeded at a density of 1×10^5^ cells per well in 6-well plates. After 24 hours, cells were transfected with siRNAs (50 nM) using Lipofectamine 3000 (Invitrogen) according to the manufacturer’s protocol. Transfection efficiency was confirmed by RT-qPCR and/or Western blot 48 hours post-transfection, showing ~70–90% decrease in mRNA levels of target genes. Detailed siRNA sequences and transfection conditions are listed in Supplementary Table S2. Experiments were repeated in triplicate to ensure reproducibility.

### RNA extraction and RT-PCR analysis

Total RNA was extracted from liver tissue using TRIzol reagent (Invitrogen, USA), and complementary DNA (cDNA) was synthesized via reverse transcription using the RT reagent (Takara, China). The ΔΔCT method was employed for quantitative analysis of gene expression, with β-actin used as the endogenous control. The amplification process included pre-denaturation at 95 °C for 15 minutes, denaturation at 94 °C for 30 seconds, annealing for 30 seconds, and extension at 72 °C for 30 seconds, followed by a total of 40 cycles. The primers used were synthesized by Sangon Biotech (Shanghai, China) (Supplementary Table S3).

### CCK8 assay

Transfection of CAL27 cells was performed using siRNAs targeting IGF2BP2, HNRNPC, TRMT61B, and NAT10 genes, employing three distinct siRNAs for each gene. This approach ensures effective gene knockdown by targeting multiple regions of each gene to enhance the likelihood of reducing their expression levels. Subsequently, the siRNA with the highest inhibition efficiency was selected for a Cell Counting Kit-8 (CCK-8) assay to assess cell viability (Beyotime Technology, China). At one, two, three, four, and five days post-transfection, 10 µL of CCK8 reagent was added to each well, and absorbance was measured at 450 nm. The mean absorbance of five wells was calculated, and each experiment was performed in triplicate. Each experiment was performed in triplicate with three independent biological replicates. The statistical power analysis for the CCK-8 assay was conducted using an estimated effect size of 1.2, a significance level (α) of 0.05, and a power (1-β) of 0.8. The analysis indicated that a sample size of at least three independent replicates per group was sufficient to detect statistically significant differences.

### Subcutaneous xenograft tumor model

Eighteen male BALB/c nude mice (5 weeks old, 15–19 g) were obtained from Shanghai SLAC Laboratory Animal Co., Ltd. and housed at Shanghai Ninth People's Hospital under institutional ethical guidelines. The study protocol was approved by the Animal Ethics Committee (No. SH9H-2024-A1118-SB). Mice were randomly divided into three groups (sh-Control, sh-NAT10, sh-IGF2BP2; n = 6 each). CAL-27 cells were transduced with lentiviral shRNAs targeting NAT10, IGF2BP2, or nonsilencing control (sh-Control). Sample size determination was based on prior tumor growth studies, with an estimated effect size of 1.5, α = 0.05, and power = 0.8, calculated using G*Power software. The analysis confirmed that six mice per group provided sufficient power to detect significant differences in tumor growth. For the experiments, 1 × 10^7^ cells were subcutaneously inoculated into the left flank of each mouse. Tumor size was measured weekly, with tumor volume calculated as 0.5 × length × width^2^, and photographs were taken to document tumor progression. After 35 days, mice were anesthetized via an intraperitoneal injection of pentobarbital sodium (50 mg/kg, Sigma-Aldrich, USA) for blood collection. Anesthesia was confirmed by the absence of a response to a toe pinch reflex. Once fully anesthetized, blood samples were obtained via cardiac puncture using a 1 mL syringe. The collected blood was centrifuged at 3,000 rpm for 15 minutes at 4 °C to separate the serum for further analysis.

Subsequently, the mice were euthanized under deep anesthesia for endpoint analysis. Each mouse received a high-dose intraperitoneal injection of pentobarbital sodium (150 mg/kg) to induce deep sedation. Death was confirmed by the cessation of respiratory and cardiac activity. Following euthanasia, tumors were carefully excised and processed for immunohistochemical analysis.

### Immunohistochemistry (IHC)

Tumor tissues were immersion-fixed in 4% paraformaldehyde for 24 hours, embedded in paraffin, and sectioned into 4 μm slices. Ki67-positive cells were detected to assess cell division within the tumor tissues. After deparaffinization and rehydration, the sections were incubated with 3% hydrogen peroxide (H_2_O_2_) in PBS for 30 minutes to inactivate endogenous peroxidases, followed by incubation in PBS containing 1% bovine serum albumin (BSA) for 30 minutes at room temperature to block nonspecific staining. The sections were then incubated with a polyclonal anti-Ki67 antibody (1:200 dilution; ab16667; Abcam, Shanghai, China) for 1 hour, followed by a goat anti-rabbit secondary antibody (1:100 dilution; ab6721; Abcam, Shanghai, China) for 1 hour at room temperature. Immunoreactivity was visualized using diaminobenzidine (DA1010; Solarbio, Shanghai, China).

### TUNEL assay

A TUNEL assay kit (C1086; Beyotime, Wuhan, China) was employed to detect apoptotic cells following the manufacturer’s instructions. Tissue sections were incubated with the TUNEL reaction mixture for 1 hour at 37 °C and subsequently rinsed three times with PBS. Images were captured at high magnification (× 400) using an inverted fluorescence microscope.

### Statistical analysis

Statistical analyses were performed using R software (version 4.0.5). For continuous variables (e.g., cell viability, tumor volume), Student’s t-test (two-group comparisons) or one-way ANOVA (≥ 3 groups) with Tukey’s post-hoc test for multiple comparisons were applied. For paired tumor vs. normal comparisons, a paired t-test was used if data were normally distributed (Shapiro–Wilk test *p* > 0.05); otherwise, the Mann–Whitney U or Kruskal–Wallis test was employed. Differential expression analysis adjusted p-values via the Benjamini–Hochberg method to control the false discovery rate (FDR) at 5%. Survival analyses utilized the Kaplan–Meier (KM) method with log-rank test for curve comparisons. The KM method was used to evaluate the survival outcomes of OSCC patients based on the RRGs signature [25] and univariate/multivariate Cox regression (via the ‘survival’ package) to evaluate risk factors (age, gender, TNM stage). Subgroup analyses in the TCGA OSCC training cohort were performed based on clinicopathological characteristics (age, sex, TNM stage). The prognostic power of our OSCC signature was compared with clinicopathological features using ROC curve analysis. The mutational status of four selected genes in TCGA datasets was determined using the cBioPortal database [26]. Additionally, the TIMER database, a freely accessible online resource [27], was used to establish the relationship between risk factors and tumor immune infiltrations. A *p*-value of ≤ 0.05 was considered statistically significant.

## Results

### Construction and evaluation of the RRGs signature

**Fig. 1:**
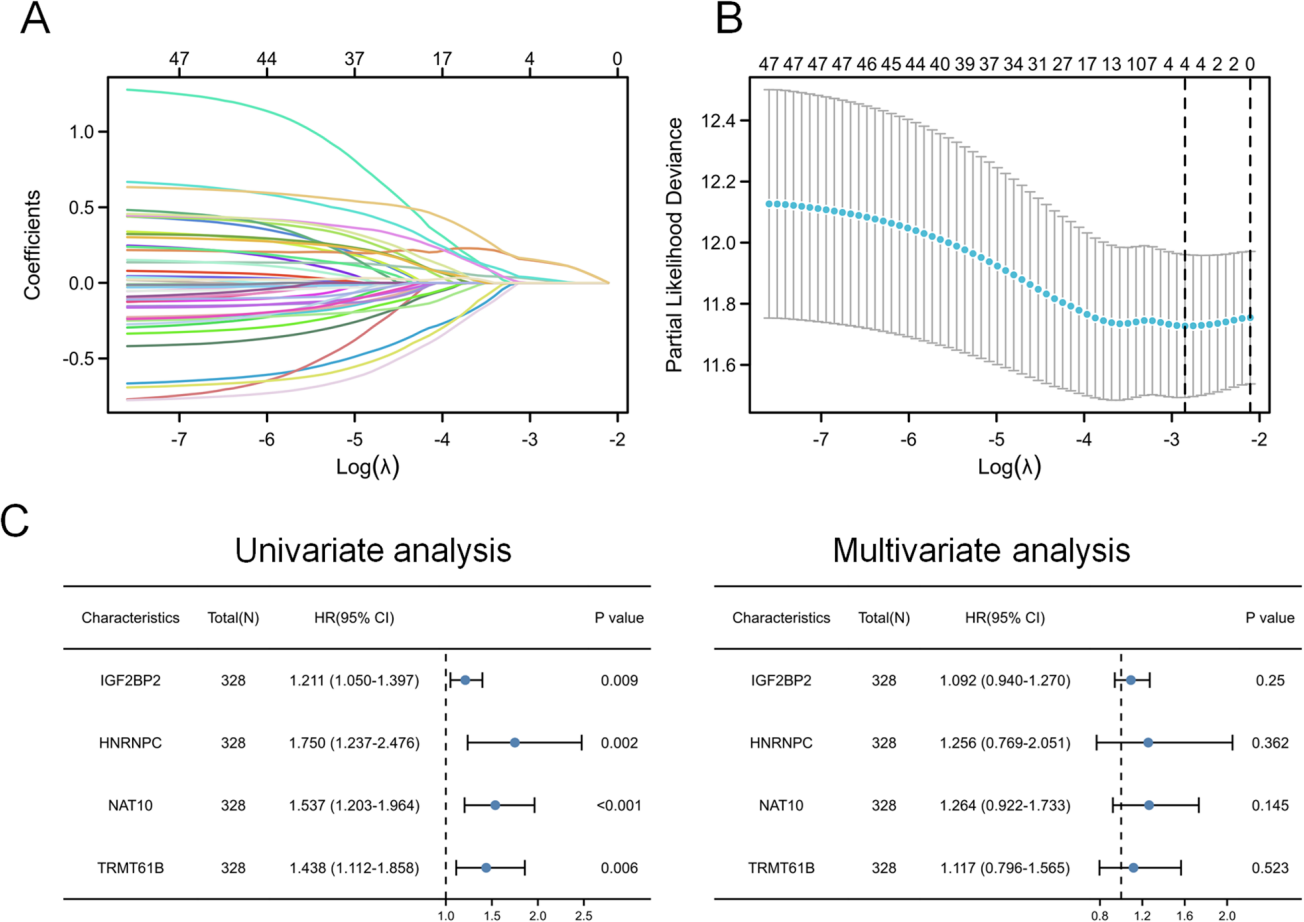
Risk model based on RRGs. **A** LASSO coefficient path plot: The horizontal axis represents the log(λ) value, and the vertical axis shows the coefficients of candidate genes. Each colored line corresponds to one gene. **B** Partial likelihood deviance plot. The blue dotted line traces the partial likelihood deviance across varying log(λ). The error bars represent the standard deviation from 10-fold cross-validation. The two vertical dashed lines mark the λ values that yield the minimum deviance (left) and the simplest model (right) within one standard error of the minimum, respectively. **C** Forest plots for univariate (left) and multivariate (right) Cox analyses. Hazard ratios (HR) with 95% confidence intervals (CIs) are shown for each of the four selected genes (IGF2BP2, HNRNPC, NAT10, and TRMT61B). Genes with an HR > 1 indicate a higher risk of poor survival when overexpressed. *p*-values reflect the significance of each gene’s correlation with overall survival in oral squamous cell carcinoma (OSCC) patients

Lasso analysis identified four out of 49 genes as significant: IGF2BP2, HNRNPC, NAT10, and TRMT61B (Fig. 1A, B). These genes were validated to have a significant correlation with OS using univariate and multivariate Cox regression analyses. To estimate each patient's survival risk, a prognostic risk model was constructed based on these four genes. The risk score is calculated as follows: Risk score = 0.088442428 × expression of IGF2BP2 + 0.228211452 × expression of HNRNPC + 0.23426084 × expression of NAT10 + 0.11020388 × expression of TRMT61B - 3.8436169. This established risk prognostic model is referred to as the OSCC prognostic model (Fig. 1C).

**Fig. 2:**
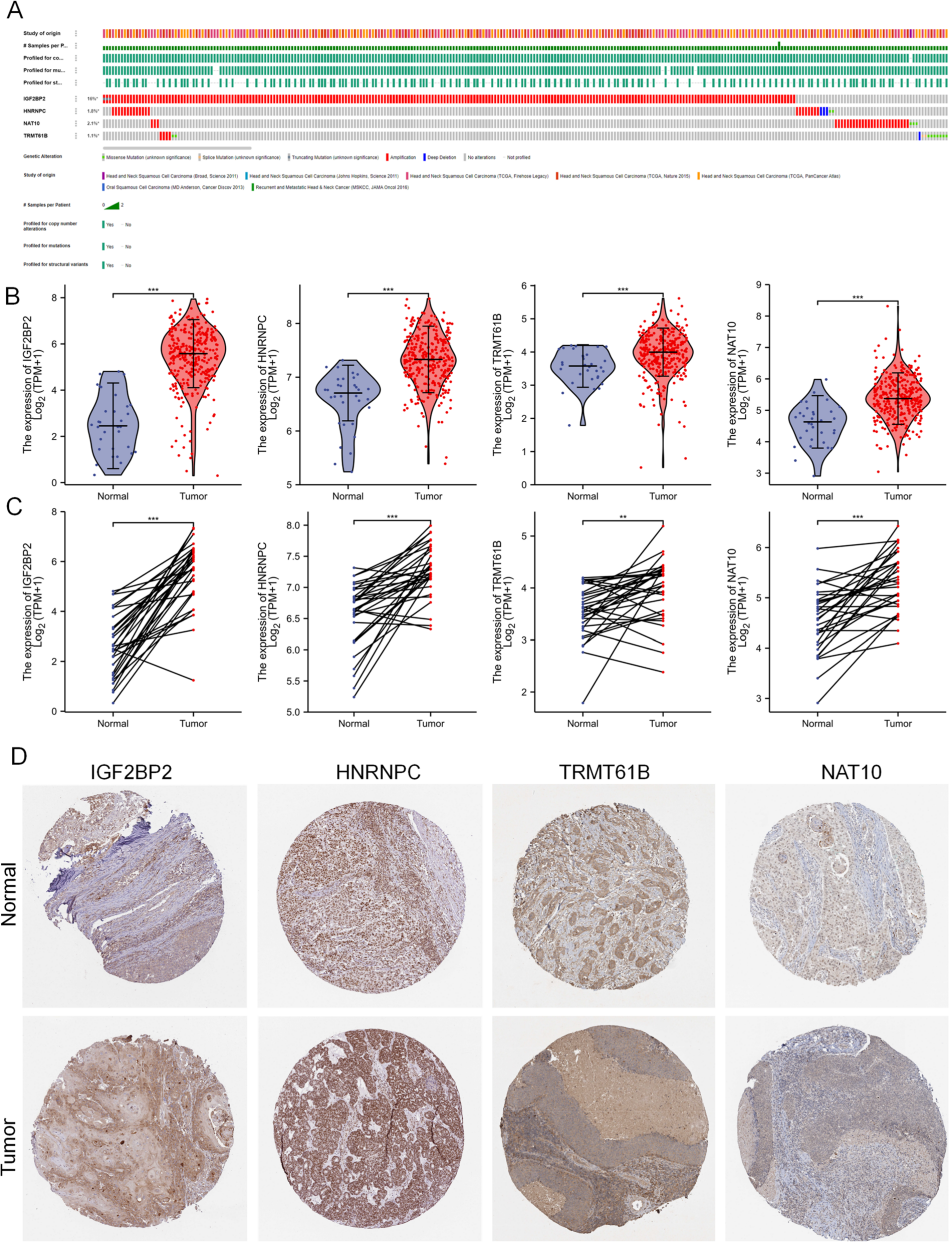
Clinical characteristics of risk scores. **A** OncoPrint of *IGF2BP2, HNRNPC, TRMT61B*, and *NAT10* using the cBioPortal database. Each column represents one patient sample, and each row corresponds to a gene. Different colors or bars indicate various genetic alterations (e.g., mutation, amplification, deep deletion). The bottom legend summarizes the frequency of each alteration type across the cohort. **B**, **C** Unpaired (**B**) and paired (**C**) differential analyses for the 4 genes between paraneoplastic and tumor tissues. The expression level of the 4 genes was higher in tumor tissues than in normal tissues. **D** Immunohistochemistry (IHC) of IGF2BP2, HNRNPC, TRMT61B, and NAT10 in normal versus tumor tissues. Representative tissue microarray images (from the Human Protein Atlas or in-house staining) show the protein localization and intensity. Brown staining indicates positive protein expression, while blue (hematoxylin) represents the nuclear counterstain. The upper panels depict normal oral mucosa, and the lower panels show corresponding OSCC tissues, confirming higher protein expression in tumor samples for most genes. Scale bars (if included) indicate the magnification used

The mutational status of these four selected genes in TCGA OSCC samples was analyzed using the cBioPortal database (Fig. 2A). The differences in the expression of these four genes between paracancerous and tumor tissues were assessed through unpaired (Fig. 2B) and paired (Fig. 2C) analyses. All four genes showed higher expression levels in tumor tissues. In addition, the Human Protein Atlas database (https://www.proteinatlas.org/) was used to assess protein expression in OSCC tissues. As shown in Fig. 2D, both mRNA and protein levels of these four genes were significantly upregulated in tumor tissues.

### Prognostic analysis of risk model and RRGs

**Fig. 3:**
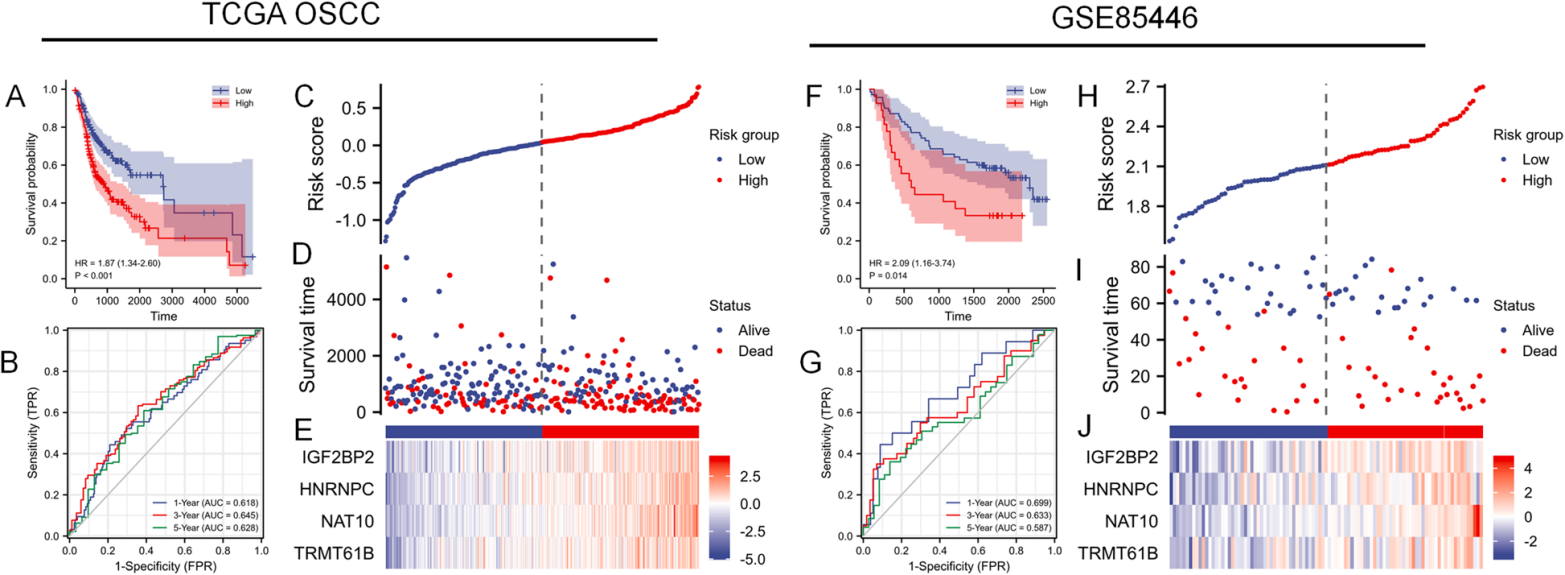
KM survival analysis, risk score assessment by the RRGs signature and time-dependent ROC curves in the TCGA OSCC cohort. **A** KM survival analysis of high- and low-risk samples of the TCGA OSCC training set. **B** ROC curves for OS of the training set. The AUC at 1-, 3-, and 5-year. **C** Risk score distribution in the training set, (**D**) survival status of the training set, and (**E**) 4 RRGs expression patterns for patients in high- and low- risk groups as determined by the four-RRGs signature in the training set. **F** KM survival analysis of high- and low-risk samples of the GSE85446 validation cohorts. **G** ROC curves for OS of the validation cohorts. The AUC at 1-, 3-, and 5-year. **H** Risk score distribution in the validation cohorts, (**I**) survival status of the validation cohorts, and (**J**) 4 RRGs expression patterns for patients in high- and low-risk groups based on the four-RRGs signature in the validation cohorts

Based on the four identified genes, the risk scores for each patient in the TCGA OSCC training set were calculated. Patients with high-risk scores were associated with higher mortality rates compared to those with low-risk scores (Fig. 3A). The area under the curve (AUC) for OS outcomes at 1-, 3-, and 5-year were 0.618, 0.645, and 0.628, respectively (Fig. 3B). Fig. 3C and D show the distributions of risk scores and survival outcomes for OSCC patients in the TCGA OSCC training set cohort. The expression levels of the four genes in high-risk and low-risk populations are displayed in the heatmap (Fig. 3E).

For external validation, we retrieved the GSE85446 dataset from the Gene Expression Omnibus (GEO) database. This dataset includes 99 OSCC samples analyzed using the Agilent-014850 Whole Human Genome Microarray 4 × 44 K G4112 F. Clinical characteristics, where available, were collected and summarized in Table 3. Compared to the TCGA cohort, the GSE85446 population showed a slightly higher proportion of early-stage tumors (42.27%), and the age distribution of patients was balanced, closely resembling that of the TCGA-OSCC cohort. We applied the same data preprocessing steps (log_2_ transformation and median-centering) for consistency. Genes missing in the GSE85446 platform were excluded. Finally, we used identical algorithms (LASSO Cox regression, Kaplan–Meier analysis) to evaluate our previously derived risk score in this validation set. In the GSE85446 cohort, the observation that high-risk score patients had a lower survival rate compared to the low-risk score group aligns with findings from the training set cohorts (Fig. 3F). For high-risk score patients, the AUCs for OS outcomes at 1-, 3-, and 5-year were 0.699, 0.633, and 0.587, respectively. Fig. 3H and Fig. 3I present the distributions of risk scores and survival outcomes for OSCC patients in the GSE85446 validation set. The expression levels of the four genes in high-risk and low-risk groups are shown in the heatmap (Fig. 3J).

**Table 3:** Clinic pathological characteristics of patients with OSCC (GSE85446)

Characteristic	No. of cases (*n* = 97)	%
Age, years (%)		
≤ 60	50	51.55
> 60	47	48.45
Gender (%)		
Female	31	31.96
Male	66	68.04
Tumor stage, n (%)		
Stage I/II	41	42.27
Stage III/IV	56	57.73

### Establishment and assessment of a nomogram

**Fig. 4:**
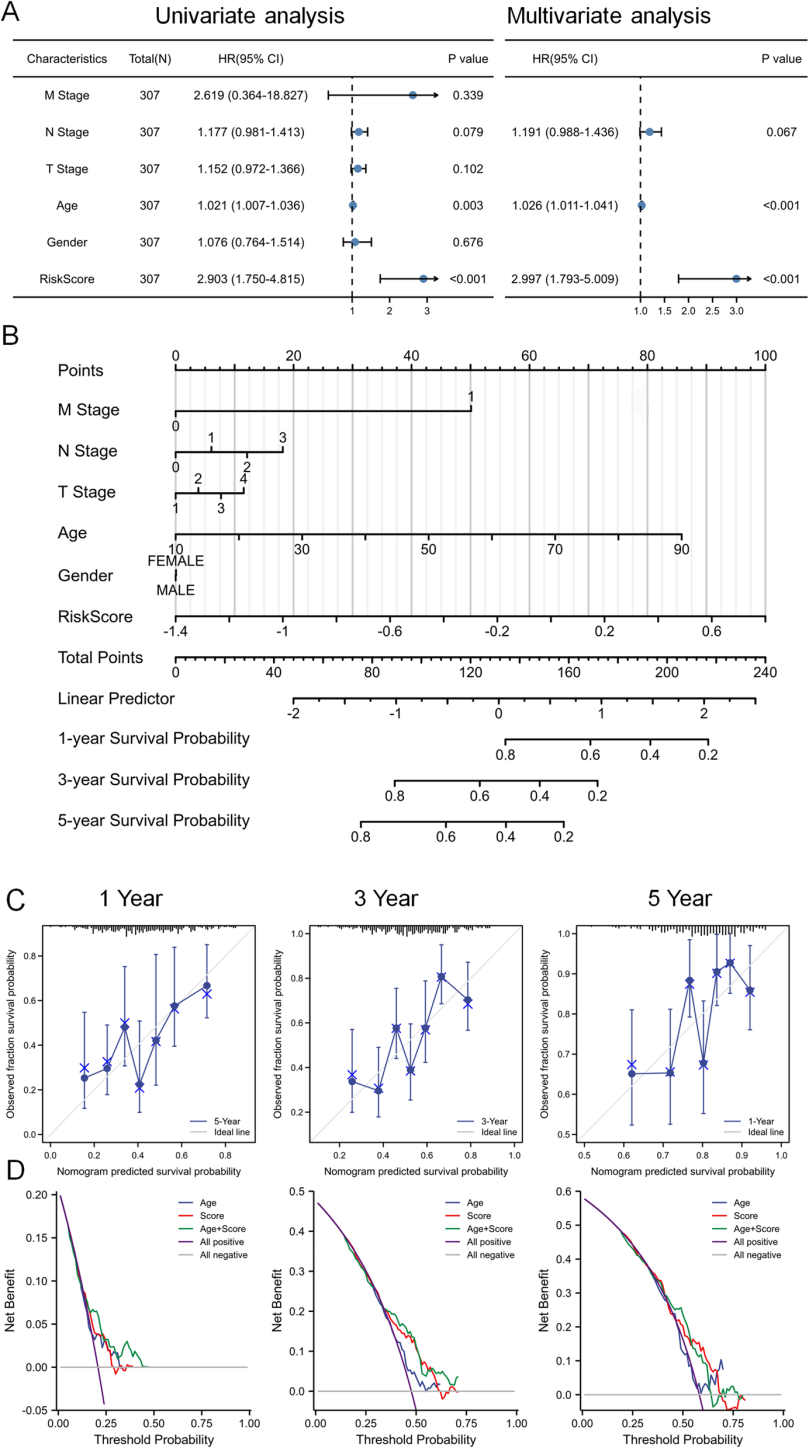
Univariate and multivariate Cox regression analysis of the 4-gene risk model, along with the establishment and assessment of a nomogram. **A** Univariate cox regression and multivariate cox regression analysis based the risk score and clinical characteristics. **B** The nomograms for predicting probabilities of OSCC patients OS. **C** The calibration curves. **D** The DCA plot

Univariate Cox regression analysis of risk factors, including age, gender, and TNM stage, revealed that the risk factor itself (hazard ratio [HR] = 2.903; 95% confidence interval [CI], 1.750−4.815, *p* < 0.001), age (HR = 1.021; 95% CI, 1.007−1.036, *p* = 0.003), and N stage (HR = 1.177; 95% CI, 0.981−1.413, *p* = 0.079) were all significantly associated with OS in OSCC patients (Fig. 4A). Multivariate Cox regression analysis indicated that the risk factor (HR = 2.997; 95% CI, 1.793−5.009) and age (HR = 1.026; 95% CI, 1.011−1.041) were independent prognostic factors. The four-gene risk model proved effective in predicting survival outcomes for OSCC patients, suggesting it may serve as a potential prognostic marker for OS in this population.

Subsequently, nomograms that incorporated variables such as risk score, age, and TNM stage were developed to create a quantitative approach for predicting the OS of OSCC patients (Fig. 4B). The nomogram demonstrated good predictive accuracy, as confirmed by the calibration curves (Fig. 4C). Furthermore, decision curve analysis (DCA) indicated that the risk factors were highly accurate in predicting 1-, 3-, and 5-year OS outcomes (Fig. 4D).

### Stratification analysis of RRGs

**Fig. 5:**
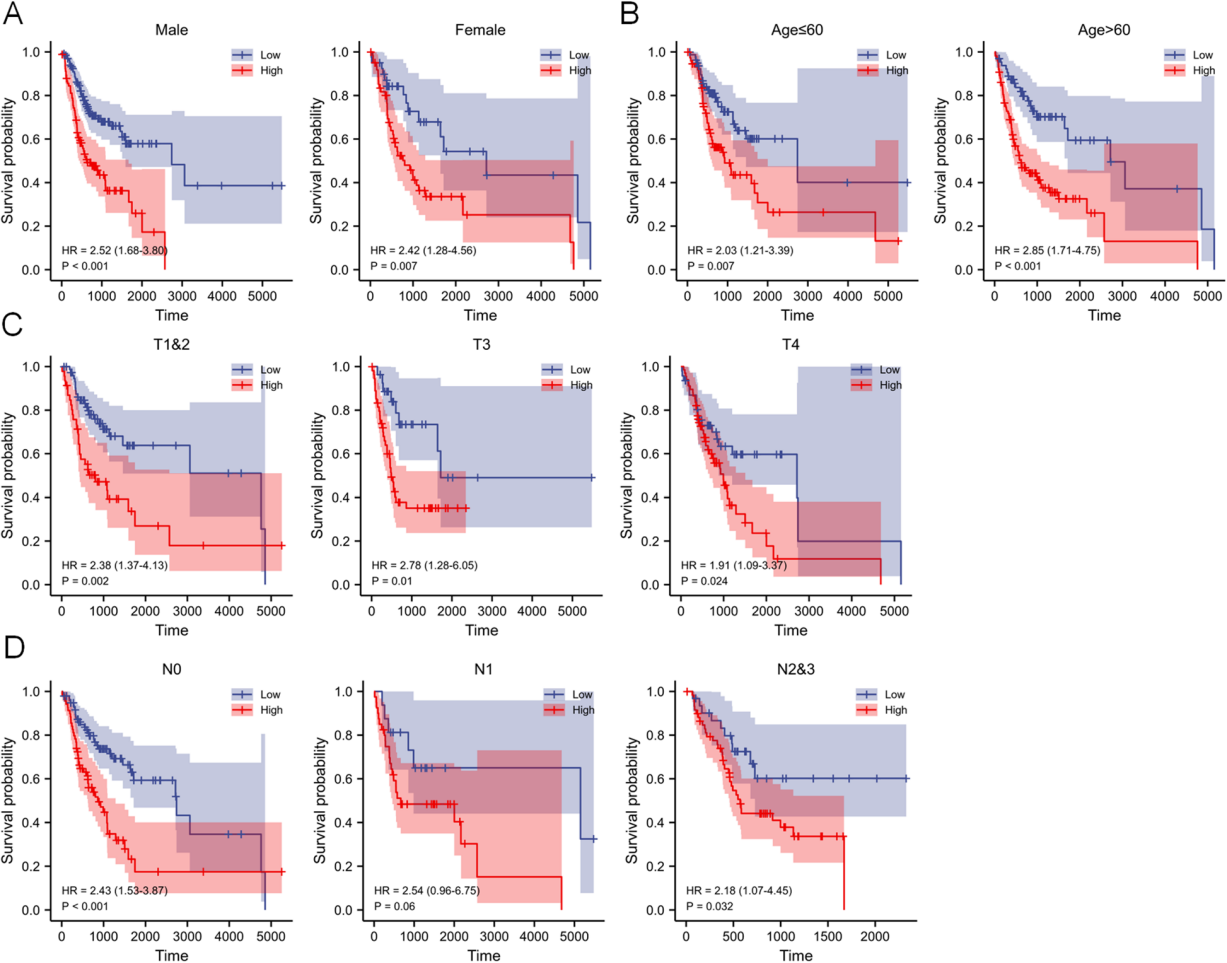
Differences in survival rates between high- and low-risk group patients stratified by clinicopathological parameters in the validated OSCC cohort. **A** Male, Female. **B** Age ≤60 y, Age >60 y. **C** T1&2, T3, T4. **D** N0, N1, N2&3. The RRGs retained their prognosis prediction value in multiple subgroups analysis of OSCC patients

Next, a subgroup analysis of relevant clinicopathological variables, including age, gender, and T/N stage, was conducted using the TCGA OSCC training set cohort. The KM curves indicated that the risk variables were stable prognostic predictors for OSCC patients when stratified by age, gender, and T/N stage. However, for T1 staging, there were no significant differences between the subgroups, possibly due to the small sample size in these categories (Fig. 5).

### Immune infiltration associated with risk score

**Fig. 6:**
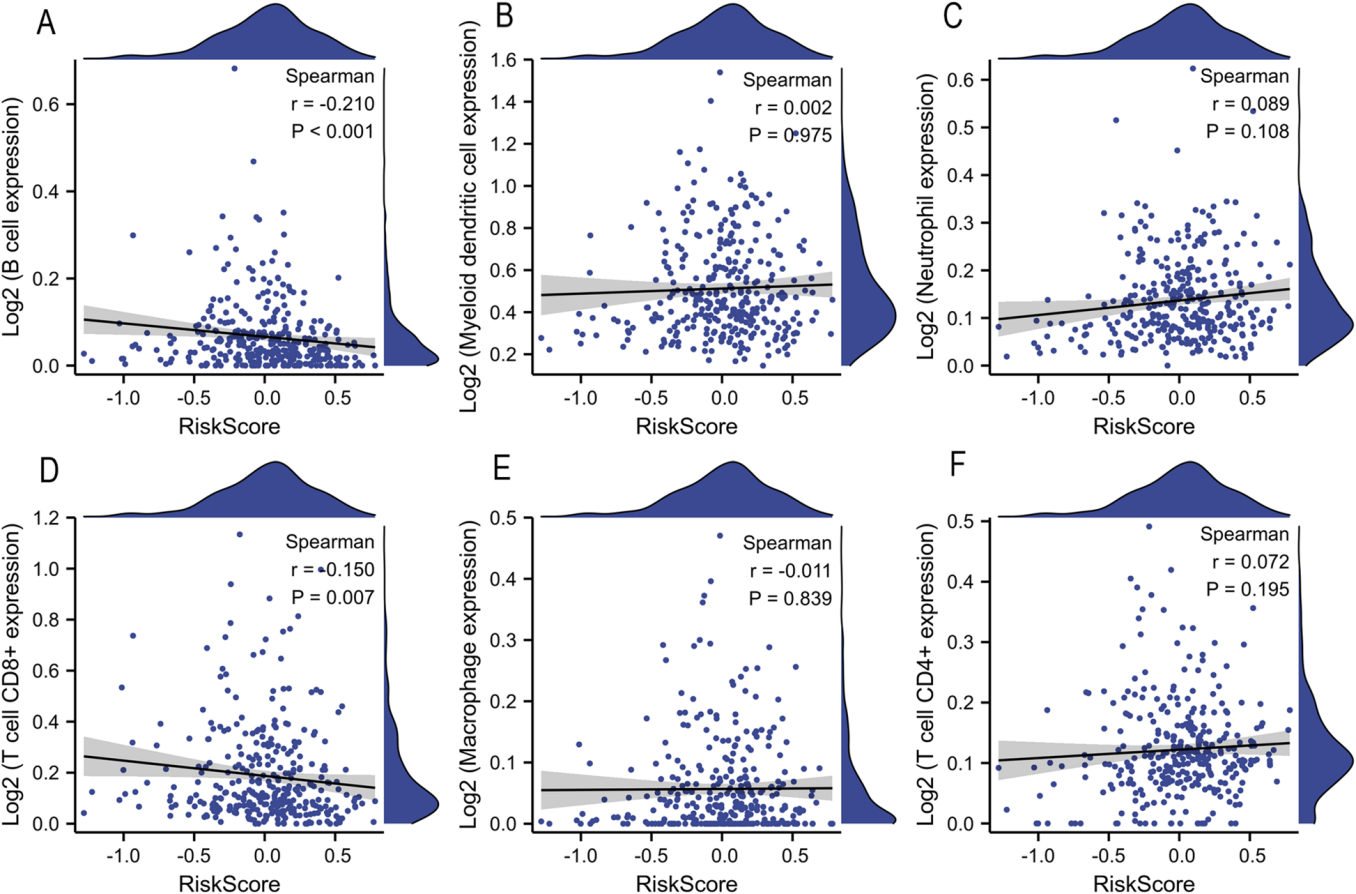
Spearman's coefficient for the relationship between risk scores and the infiltration level of 6 immune cell types. **A** B cell, (**B**) Myeloid dendritic cell, (**C**) neutrophil, (**D**) CD8^+^T cell, (**E**) macrophage, and (**F**) CD4^+^T cell. The blue line in each graph corresponds to the linear model and represents the proportionate trend of tumor-infiltrating immune cell score (TICS) and risk score. The gray shading surrounding the blue line represent the 95% CI

TIMER was used to investigate the relationship between risk scores and tumor immune infiltration. To assess the effects of the four-RRGs signature on the OSCC immune microenvironment, the infiltration levels of six immune cell types were analyzed. We found significant negative correlations between the risk score and the infiltrations of B cells and CD8^+^ T cells (*p* = 0.007; Fig 6). This finding suggests that the four-RRGs signature is associated with the immune microenvironment in OSCC.

### GSEA results and KEGG/GO enrichment

**Fig. 7:**
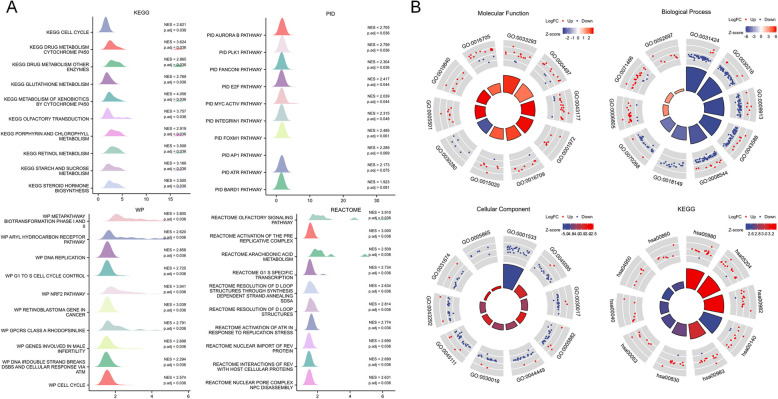
GSEA and KEGG/GO enrichment analysis showing the potential biological functions and signaling pathways of the 4 genes (IGF2BP2, HNRNPC, TRMT61B, and NAT10). **A** GESA analysis revealed that the 4 genes were significantly in critical biological functions and signal pathways which were correlated with cell circulation, drug metabolism, and glutathione metabolism. **B** The result of KEGG/GO analyses indicated that the 4 genes were primarily involved in critical biological functions and signaling pathways involved in drug metabolism, skin development, epidermal growth, and keratinocyte differentiation

The 328 OSCC patients in the TCGA OSCC database were divided into high- and low-risk groups based on their risk scores (Fig. 7A). GSEA was used to evaluate differentially expressed genes (DEGs) in these groups (Supplementary Table S4). The DEGs in the high-risk score group were enriched in pathways related to cell circulation, drug metabolism, and glutathione metabolism. Additionally, KEGG/GO analysis (Fig. 7B) indicated that DEGs in the high-risk score group were associated with drug metabolism, skin development, epidermal growth, and keratinocyte differentiation (Supplementary Table S5).

### NAT10 and IGF2BP2 promotes proliferation in vitro and in vivo

**Fig. 8:**
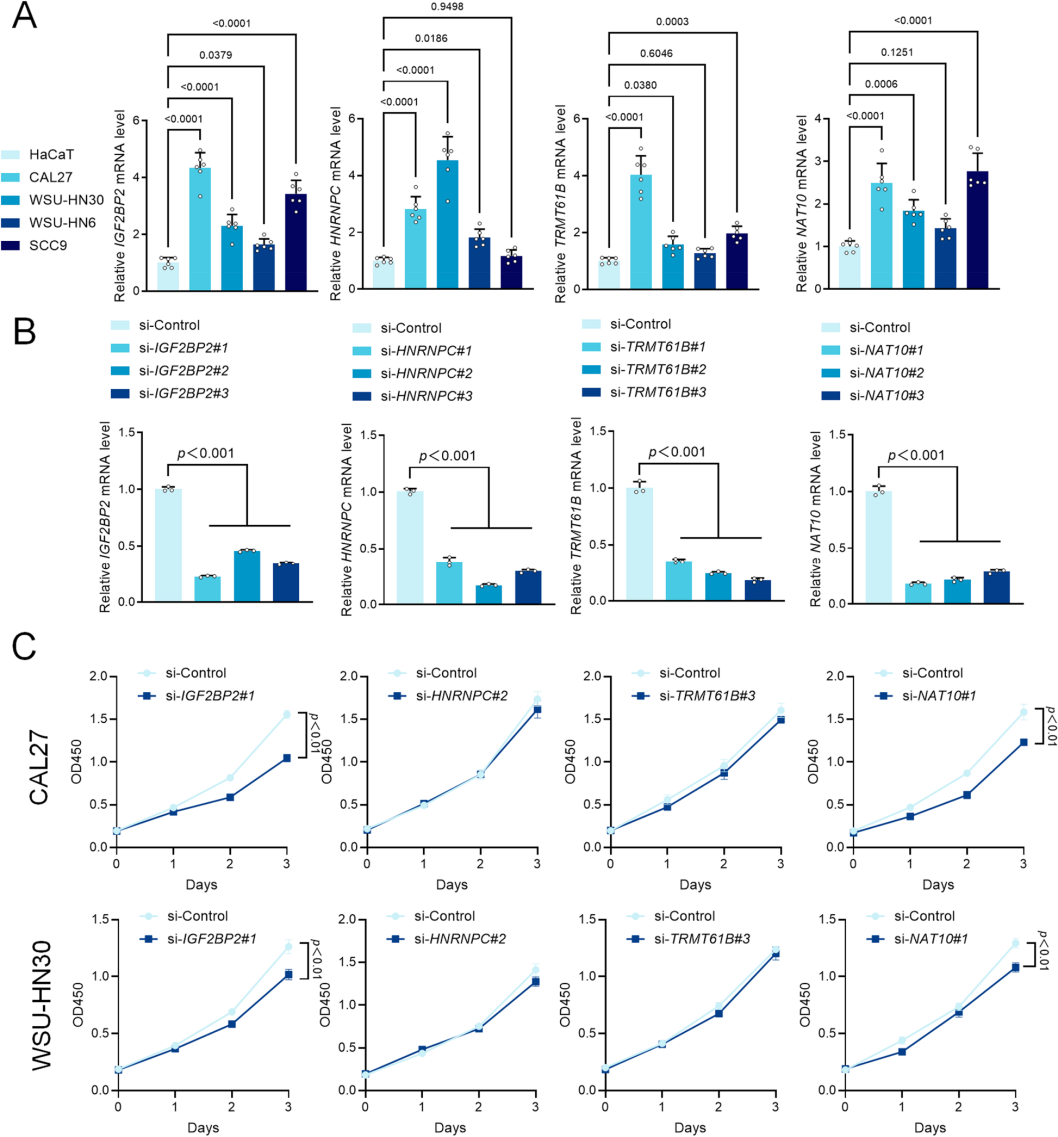
Inhibition of NAT10 and IGF2BP2 expression using siRNA inhibits proliferation in vitro. **A** The mRNA level of IGF2BP2, HNRNPC, NAT10, and TRMT61B in OSCC cell lines CAL27, WSU-HN30, WSU-HN6, SCC9, and HaCaT detected by RT-PCR. **B** IGF2BP2, HNRNPC, NAT10, and TRMT61B expression was decreased by IGF2BP2, HNRNPC, NAT10, and TRMT61B siRNAs in CAL27. **C** The proliferation potential of cells was assayed by CCK8 in CAL27 and WSU-HN30, and inhibition of NAT10 and IGF2BP2 suppressed the proliferation of CAL27 and WSU-HN30 cells

The expression levels of these four genes were evaluated across different cell lines. As shown in Fig 8A, the mRNA levels of IGF2BP2 were higher in the OSCC cell lines CAL27, WSU-HN30, WSU-HN6, and SCC9 compared to human epidermal keratinocyte cell line HaCaT. Additionally, the mRNA levels of HNRNPC were significantly increased in CAL27, WSU-HN30, and WSU-HN6. The mRNA levels of TRMT61B and NAT10 were significantly elevated in CAL27, WSU-HN30, and SCC9 (*p* < 0.05).

To ascertain the effects of IGF2BP2, HNRNPC, TRMT61B, and NAT10 on survival rates of CAL27 and WSU-HN30 cells, siRNA was used to individually inhibit the expression of each of these four genes. We transfected siRNAs of these four genes in CAL27 cells with 3 siRNAs per gene. RT-PCR assay showed that the expression of IGF2BP2, HNRNPC, TRMT61B, and NAT10 were decreased in CAL27 (*p* < 0.001) (Fig. 8B). Next, cell proliferation was detected by CCK8 assay, and the results indicated that inhibition of NAT10 and IGF2BP2 suppressed the proliferation of CAL27 and WSU-HN30 cells (*p* < 0.01) (Fig. 8C). These data demonstrate that NAT10 and IGF2BP2 participates in regulating the proliferation of OSCC cell lines.

**Fig. 9:**
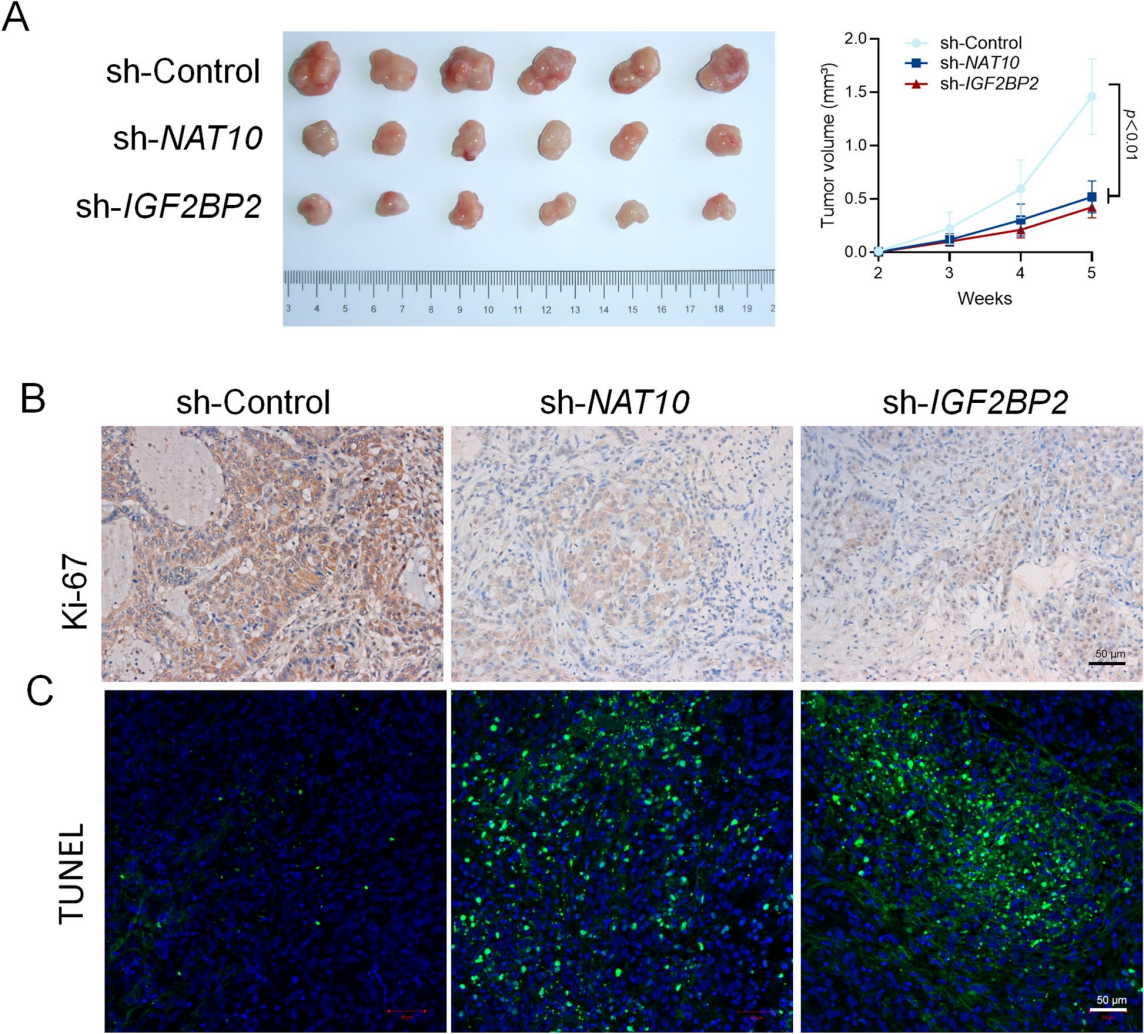
Inhibition of NAT10 and IGF2BP2 expression using shRNA inhibits proliferation in vivo. **A** The sh-NAT10 and sh-IGF2BP2 CAL27 subcutaneous tumors showed smaller size compared with control tumors. **B** IHC staining in the mice subcutaneous tumors. sh-NAT10 and sh-IGF2BP2 CAL27 tumor showed lower staining of Ki-67 compared with control CAL27 tumors. Scale bar: 50 μm. **C** Analysis of TUNEL-positive cells in the mice subcutaneous tumors. sh-NAT10 and sh-IGF2BP2 CAL27 tumor showed higher staining of TUNEL compared with control CAL27 tumors. Scale bar: 50 μm

In vivo experiments involving the subcutaneous implantation of sh-Control, sh-NAT10, and sh-IGF2BP2 CAL27 cells in nude mice demonstrated a significant decrease in tumor size with the interference of NAT10 and IGF2BP2 (*p* < 0.001) (Fig. 9A). Analysis of Ki-67 expression in tumors through IHC staining showed that Ki-67 was downregulated in both sh-NAT10 and sh-IGF2BP2 CAL27 subcutaneous tumors, indicating impaired proliferation in these tumors (Fig. 9B). Additionally, TUNEL staining revealed that transfection with sh-NAT10 and sh-IGF2BP2 significantly increased the number of TUNEL-positive cells in the mice subcutaneous tumors (Fig. 9C). These findings collectively indicate that NAT10 and IGF2BP2 play a crucial role in promoting OSCC proliferation.

## Discussion

Multigene analyses are pivotal for tumor characterization and clinical decision-making[28, 29]. Dysregulated RNA modification pathways—including methylation and acetylation—have emerged as therapeutic targets in cancer[30], with their epigenetic alterations offering reversible therapeutic opportunities distinct from genetic mutations[31]. However, no prognostic model integrating m6A/m1A/m5C/m7G/ac4C RRGs to predict overall survival in OSCC has been established.

This study analyzed RRG expression and survival associations in TCGA OSCC data, constructing a four-gene risk signature (IGF2BP2, HNRNPC, TRMT61B, NAT10) as an independent OS predictor. The model outperformed conventional clinical traits, achieving a 5-year AUC of 0.628—superior to prior studies (Wang et al.: 0.607 [22] and Jiang et al.: 0.602) [32]). *In vitro/in vivo* experiments confirmed that RRG inhibition suppresses OSCC cell proliferation.

IGF2BP2 (an m6A reader), HNRNPC (an m6A reader), TRMT61B (an m1A writer), and NAT10 (an ac4C writer) were found to be highly expressed in OSCC tissues and significantly associated with age, gender, and T/N stage. A review of the reports revealed that these genes are critical molecules in several tumors’ development, progression, and metastasis. Among these, IGF2BP2, HNRNPC, NAT10, and TRMT61B were identified as biomarkers related to RNA modifications in OSCC and appear to serve as potential candidates for early diagnosis and prognostic assessments. Insulin-like growth factor 2 (IGF2) mRNA-binding proteins (IGF2BPs) are a large family of RNA-binding proteins (RBPs) that act as readers [33]. IGF2BP2, a member of the IGF2BPs family, regulates cellular metabolism by acting as a post-transcriptional regulator of mRNA localization, stability, and translation. IGF2BP2 could bind to and stabilize specific mRNA in tumors in an m6 A-dependent manner, influencing the proliferation of cancer cell lines in vitro, as well as tumorigenicity in vivo [34, 35]. Its abnormal expression is associated with cancer development [36], and elevated levels of IGF2BP2 in pancreatic cancer [35, 37], colorectal cancer [38], or esophageal squamous cell carcinoma [34] have been linked to poor prognostic outcomes. Furthermore, high expression levels of IGF2BP2 in OSCC not only promote disease progression but also correlate with cell proliferation, metastasis, and tumor immune cell infiltration [39, 40], which aligns with our findings. Our research results indicate that reducing IGF2BP2 expression inhibits proliferation of OSCC cell lines in vitro and influenced tumorigenicity in vivo. Small-molecule inhibitors or RNA-based therapies (e.g., antisense oligonucleotides) could be explored to downregulate IGF2BP2 expression or block its function [38, 41]. N-acetyltransferase 10 (NAT10) is an enzyme involved in acetylation modifications, and plays a critical role in this process [42–44]. By promoting hTERT transcription, NAT10 enhances telomerase activity [45]. Its expression is significantly associated with tumor immune infiltration and tumor development, which can enhance tumor cell growth [46–49]. NAT10 expression is notably higher in cancer tissues than in normal tissues, with elevated levels linked to poor prognostic outcomes in head and neck squamous cell carcinoma, hepatocellular carcinoma, and pancreatic cancer [43, 50]. In OSCC, we found that NAT10 is highly expressed and significantly correlated with tumor immune infiltration. Additionally, inhibiting NAT10 expression also reduces proliferation of OSCC cell lines in vitro and influenced tumorigenicity in vivo, further supporting the previous findings by Liu et al. [51]. Pharmacological inhibitors of NAT10, such as remodelin, could be repurposed or optimized for OSCC treatment [46].

HNRNPC, a member of the heterogeneous nuclear ribonucleoprotein (HNRNP) family, plays a crucial role in cellular nucleic acid metabolism and is significantly involved in cancer development [52]. Huang et al. [12] reported that HNRNPC is a potential independent prognostic biomarker for OSCC, and its overexpression enhances OSCC carcinogenicity via epithelial-mesenchymal transition (EMT). The study by Zhu et al. [53] revealed the importance of the CYTOR-HNRNPC-ZEB1 axis in regulating mitochondrial metabolism and glycolysis in oral cancer cells. CRISPR-based approaches could be employed to silence HNRNPC and impair tumor metabolism [54]. The expression of TRMT61B in OSCC has not been extensively documented. TRMT61B is a methyltransferase responsible for 1-methyladenosine modifications in human mitochondrial tRNAs at position 58, which is essential for the stability and function of tRNAs required for respiratory factor translation [55]. TRMT61B has been shown to be differentially expressed in various tumor types, including gastric cancer [56] and highly aneuploid tumors [57], and is associated with poor clinical prognosis in gastric cancer patients[56]. Martín et al. reported that depletion of TRMT61B suppresses the expression of multiple mitochondria-encoded proteins and inhibits mitochondrial functions, indicating that TRMT61B may serve as a novel biomarker for targeting highly aneuploid tumors [57]. In this study, high expression levels of TRMT61B in OSCC patients were associated with low OS and poor prognostic outcomes, suggesting that it may be a potential novel biomarker and treatment target for OSCC. Small-molecule inhibitors targeting TRMT61B’s methyltransferase activity could disrupt mitochondrial function and suppress tumor growth, but further studies are needed to evaluate their therapeutic efficacy. These findings further support the crucial roles of IGF2BP2, NAT10, HNRNPC, and TRMT61B in OSCC development and provide additional evidence for the stability and reliability of the IGF2BP2/NAT10/HNRNPC/TRMT61B-based risk signature.

Furthermore, the tumor microenvironment (TME) has a synergistic role in cancer development, and RNA modifications are involved in regulating this microenvironment [58, 59]. There is accumulating evidence that TME plays a crucial role in facilitating tumor growth and invasion, while also helping to protect tumors from the host immune system [60, 61]. Tumor cells can evade the host immune system by inhibiting their own immunogenicity and secreting immunosuppressive signals, which in turn promotes tumorigenesis, progression, and metastasis [62]. In light of this, the potential impact of immune activity on OSCC was investigated based on the RRGs risk model. In our study, TIMER analysis revealed a strong association between OSCC and tumor immune infiltration, particularly showing a significant negative relationship with CD8^+^ T cells and B cells, which are typically associated with poor prognostic outcomes in OSCC, consistent with previous findings [24, 63]. They found that the distribution of CD8^+^ T cells and B cells contributed to the tumor's ability to evade the immune response, which also appears to account for the divergent prognoses in these patients. These findings emphasize the immunosuppressive characteristics of OSCC, suggesting that immune evasion may be a key mechanism underlying its aggressive behavior. Moreover, we also performed GSEA and KEGG/GO pathway analyses to identify key biological functions and pathways associated with cancer. GSEA revealed significant enrichment of the RRGs in pathways related to cell circulation, drug metabolism, and glutathione metabolism pathways. KEGG/GO analysis also indicated that these RRGs were associated with drug metabolism, skin development, epidermal growth, and keratinocyte differentiation. Notably, mutations or abnormal expressions of the identified biological processes and pathways have been implicated in [64, 65] and many other cancers [66–70], particularly in the epidermal growth and keratinocyte differentiation pathways. These findings suggest that dysregulation of these pathways may contribute to the pathogenesis of OSCC and provide a strong foundation for further exploration into their molecular mechanisms.

This study has several strengths. First, we established a prognostic signature was established based on m6A/m1A/m5C/m7G/ac4C RRGs, and its applicability was confirmed using multiple datasets and functional validation. Second, this signature has demonstrated reliability and stability for estimating the prognosis of OSCC patients. Additionally, the genes associated with this signature play significant roles in OSCC development and may serve as potential biomarkers for prognosis. However, there are limitations to this study. The overrepresentation of Western cohorts in TCGA causes population bias in the model, necessitating validation in ethnically diverse populations, particularly Asian cohorts. While RT-PCR validated gene expression patterns, further confirmation using techniques like immunohistochemistry (IHC) and RNA sequencing in independent cohorts is essential to strengthen its clinical utility. Additionally, the integration of this gene signature with conventional TNM staging and histopathological features remains unexplored, highlighting the need for future studies leveraging machine-learning platforms to combine molecular signatures with clinical data for enhanced diagnostic precision. Although the genes show prognostic value, their potential as therapeutic targets require further investigation, particularly through studies exploring targeted therapies such as RNA-modifying enzyme inhibitors. The retrospective design and reliance on TCGA data may also introduce selection bias, underscoring the importance of larger prospective studies and subgroup analyses to refine the model’s accuracy and applicability. Addressing these limitations through rigorous validation and translational research could enable this model to become a cornerstone for precision oncology strategies in OSCC, facilitating tailored prognostication and individualized treatment.

## Conclusion

Our findings reveal that IGF2BP2, HNRNPC, NAT10, and TRMT61B play pivotal roles in OSCC progression and prognosis. The four-gene risk model may serve as a promising tool for patient stratification, while also offering potential therapeutic targets for future interventions. Prospective, multi-center studies are warranted to validate these results and to explore targeted therapies based on dysregulated RNA modification pathways in OSCC.

## Supplementary Information

Supplementary Material 1: Supplementary Table S1: Clinicopathological characteristics of OSCC patients.

Supplementary Material 2: Supplementary Table S2: List of siRNA sequences.

Supplementary Material 3: Supplementary Table S3: The primer sequences used in the RT-PCR.

Supplementary Material 4: Supplementary Table S4: Differentially expressed genes between high- and low-risk groups.

Supplementary Material 5: Supplementary Table S5: Commentary for Fig. 7B.

## Data Availability

All data produced or analyzed in this study (and its supplementary information files) are provided in the article. All datasets used in this study are publicly accessible: TCGA website ( https://portal.gdc.cancer.gov/ ). Any inquiries should be addressed to the corresponding author/s.

## Abbreviations

OSCC: oral squamous cell carcinoma
RRGs: RNA modification-related genes
m6A: N6-methyladenosine
m5C: 5-methylcytosine
m1A: N1-methyladenosine
m7G: 7-methylguanosine
ac4C: N4-acetylcytidine
IGF2BP2: insulin-like growth factor 2 mRNA-binding protein 2
TRMT61B: human tRNA methyltransferase 61B
HNRNPC: heterogeneous nuclear ribonucleoprotein C
HNRNPs: heterogeneous nuclear ribonucleoproteins
NAT10: N-acetyltransferase 10
TCGA: the Cancer Genome Atlas
KM: Kaplan-Meier
ROC: receiver operating characteristic curve
AUC: area under the curve
DCA: the decision curve analysis
GSEA: gene set enrichment analysis
KEGG: Kyoto encyclopedia of genes and genomes
GO: gene ontology
OS: overall survival
PC: pancreatic cancer
HC: hepatocellular carcinoma
HNSCC: head and neck squamous cell carcinoma
EMT: the epithelial-mesenchymal transition
