# Supplementary material for: Bioinformatics identification and validation of m6A/m1A/m5C/m7G/ac4 C-modified genes in oral squamous cell carcinoma
Source: BMC Cancer. 2025 Jul 1;25:1055. doi: 10.1186/s12885-025-14216-7 (PMC12211329; doi:10.1186/s12885-025-14216-7)
Supplement: Supplementary file 2 — Supplementary Material 2: Supplementary Table S2: List of siRNA sequences. [file 12885_2025_14216_MOESM2_ESM.docx]

**Supplementary Table S2.** List of siRNA sequences

| **Gene** | **Sense (5' → 3')** | **Antisense (5' → 3')** |
| --- | --- | --- |
| IGF2BP2#1 | GCAUAUACAACCCGGAAAGAAdTdT | UUCUUUCCGGGUUGUAUAUGCdTdT |
| IGF2BP2#2 | CGGAUCUUUGGGAAACUGAAAdTdT | UUUCAGUUUCCCAAAGAUCCGdTdT |
| IGF2BP2#3 | AAUGCUGAUGCUGAUGCUGAUdTdT | AUCAGCAUCAGCAUCAGCAUAdTdT |
| HNRNPC#1 | GGAUGAUGAUGAUGAUGAUTTdTdT | AUCAUCUCAUCUCAUCUCAUCdTdT |
| HNRNPC#2 | CCAUGAUGAUGAUGAUGAUTTdTdT | AUCAUCUCAUCUCAUCUCAUGdTdT |
| HNRNPC#3 | UGAUGAUGAUGAUGAUGAUTTdTdT | AUCAUCUCAUCUCAUCUCAUCdTdT |
| NAT10#1 | UGGUGUACUACAUUGGUGUTTdTdT | ACACCAUGUAGUACACCACAdTdT |
| NAT10#2 | CCAGUACUACAUUGGUGUATTdTdT | UACACCAUGUAGUACUGGUGdTdT |
| NAT10#3 | GGUCAUCGAGUACUACAUUTTdTdT | AAUGUAGUACUCGGAUGACCdTdT |
| TRMT61B#1 | GGUCAUCGAGUACUACAUUTTdTdT | AAUGUAGUACUCGGAUGACCdTdT |
| TRMT61B#2 | CCAGUACUACAUUGGUGUATTdTdT | UACACCAUGUAGUACUGGUGdTdT |
| TRMT61B#3 | UGGUGUACUACAUUGGUGUTTdTdT | ACACCAUGUAGUACACCACAdTdT |
| Control | UUCUCCGAACGUGUCACGUTTdTdT | ACGUGACACGUUCGGAGAATdTdT |
