# Supplementary material for: Bioinformatics identification and validation of m6A/m1A/m5C/m7G/ac4 C-modified genes in oral squamous cell carcinoma
Source: BMC Cancer. 2025 Jul 1;25:1055. doi: 10.1186/s12885-025-14216-7 (PMC12211329; doi:10.1186/s12885-025-14216-7)
Supplement: Supplementary file 3 — Supplementary Material 3: Supplementary Table S3: The primer sequences used in the RT-PCR. [file 12885_2025_14216_MOESM3_ESM.docx]

**Supplementary Table S3.** The primer sequences used in the RT-PCR.

| Genes | Forward | Reverse |
| --- | --- | --- |
| IGF2BP2 | AGCTAAGCGGGCATCAGTTTG | CCGCAGCGGGAAATCAATCT |
| HNRNPC | GCCAGCAACGTTACCAACAA | TGAACAGAGCAGCCCACAAT |
| TRMT61B | AGGGGCTCGGAACCAATTC | GCAGGTCTCTAGGCGAGGA |
| NAT10 | ATAGCAGCCACAAACATTCGC | ACACACATGCCGAAGGTATTG |
| β-actin | CATGTACGTTGCTATCCAGGC | CTCCTTAATGTCACGCACGAT |
